# Dispensary Attendances

**Published:** 1915-07-31

**Authors:** 


### Dispensary Attendances.

*To the Editor of* THE HOSPITAL.

SIR,—THE HOSPITAL this week raises the question as to why there is a falling-off both in dispensary attendances by tuberculous persons, and also in the applications for sanatorium benefit to the Insurance Committees.

May one venture to suggest that the primary cause lies in the fact that trade is, and has been for some time, exceptionally good? Especially is it so in two trades which come to one's mind, largely followed by tuberculous persons—namely, tailoring and bootmaking.

Speaking from many years' experience of sanatorium administration, it has always been seen that the length of the waiting list is in direct ratio to the state of the labour market. There is no doubt that the condition of trade, and consequently of wages, has its effect upon the nourishment of these workers, and thus on their disease. One imagines, secondly, that the Insurance Committees are not expending more than they can help at a time like this in providing sanatorium treatment for the insured.—Believe me, yours faithfully, SANATORIUM OFFICER.

